# Adult attachment style as a risk factor for maternal postnatal depression: a systematic review

**DOI:** 10.1186/s40359-014-0056-x

**Published:** 2014-12-18

**Authors:** Nasir Warfa, Melissa Harper, Giampaolo Nicolais, Kamaldeep Bhui

**Affiliations:** Centre for Psychiatry, Wolfson Institute of Preventive Medicine, Old Anatomy Building, Barts and The London School of Medicine & Dentistry, Queen Mary University of London, Charterhouse Square, London, EC1M 6BQ UK; Sapienza University, Piazzale Aldo Moro, 5, 00185 Rome, Italy; Faculty Member, HPRT, Department of Continuing Medical Education, Harvard Medical School, 22 Putnam Avenue, Cambridge, MA, Boston 02139 USA

**Keywords:** Postnatal depression, Maternal depression, Maternal mental health, Attachment theory, Adult attachment style, Review systematic

## Abstract

**Background:**

Postnatal depression (PND) is an important health problem of global relevance for maternal health and impacts on the health and wellbeing of the child over the life-course. Multinational data is hard to locate, the economic burden of PND on health care systems have been calculated in several countries, including Canada and in the UK. In Canada, health and social care costs for a mother with PND were found to be just over twice that of mothers with no mental illness. The extra community care cost for women with PND living in the UK was found to be £35.7 million per year.

**Method:**

We carried out a systematic search to the literature to investigate the associations between attachment style and PND, using meta-narrative analysis methods, reporting statistical data and life narratives. The following databases were searched: PsycInfo, PsycExtra Web of Science, The Cochrane Library and Pubmed. We focused on research papers that examined adult attachment styles and PND, and published between 1991 and 2013. *We included any papers showing relationship between maternal adult attachment and PND*. Out of 353 papers, 20 met the study inclusion criteria, representing a total of 2306 participants. Data from these 20 studies was extracted by means of a data extraction table.

**Results:**

We found that attachment and PND share a common aetiology and that ‘insecure adult attachment style’ is an additional risk factor for PND. Of the insecure adult attachment styles, anxious styles were found to be associated with PND symptoms more frequently than avoidant or dismissing styles of attachment.

**Conclusion:**

More comprehensive longitudinal research would be crucial to examine possible cause-effect associations between adult attachment style (as an intergenerational construct and risk factor) and PND (as an important maternal mental health), with new screening and interventions being essential for alleviating the suffering and consequences of PND. If more is understood about the risk profile of a new or prospective mother, more can be done to prevent the illness trajectory (PND); as well as making existing screening measures and treatment options more widely available.

**Electronic supplementary material:**

The online version of this article (doi:10.1186/s40359-014-0056-x) contains supplementary material, which is available to authorized users.

## Background

Postnatal Depression, PND, (also called Postpartum Depression) is a major depressive disorder or episode with a “*Postpartum Onset Specifier*” (APA 2000, pp422), sharing the same diagnostic criteria as depression. The ICD-10 classifies it as a “*Mild mental and behavioural disorder associated with the peurperium, not elsewhere classified*”. These different definitions refer PND development from four weeks to one year.

The prevalence of PND in western countries varies from 13% to 19.2% (O’hara and Swain 1996; Josefsson et al. 2001; Huang and Mathers 2001; Gavin et al. 2005). In low and middle-income countries (LMIC), pooled estimates of prevalence rates are reported to be 19.8% (Fisher et al. 2012) and range between 3.5% to 63% (N = 76). The epidemiology of PND is fairly well researched with many empirically supported risk factors giving rise to a complex bio-psychosocial model for its development. Biological risk factors include diet, hormonal changes, childbirth complications and genetic disposition (Starr et al. 2013; Rowe et al. 2013 Bhandari et al. 2012). The strongest psychosocial predictors of depression are depression and/or anxiety in pregnancy, stressful life events, low social support and previous history of mental illness (O’Higgins et al. 2013; Conde et al. 2011; Robertson et al. 2004). Other widely supported psychosocial risk factors include low self-esteem, adjustment problems in the transition to parenthood, vulnerable personality traits (particularly neuroticism), maternal childhood problems (including abuse), low socio-economic status, and relationship discord (NICE 2007; O’hara and Swain 1996; Beck 2001; Robertson et al. 2004).

### Attachment theory and postnatal depression

Attachment behaviour can be defined as any behaviour that one elicits with the goal of attracting a defined person deemed better able to deal with the world, to move closer to and to offer care and comfort (Ainsworth 1978; Bowlby 1988). This behavioural system is organised internally in early life reflecting the child’s experience of adapting to his or her caregivers with specific care seeking or soothing behaviours (Ainsworth 1979). In Bowlby’s conceptualisation, all humans are born with this biologically pre-wired disposition that allows us to seek protection when in trouble. The attachment system, one of the main motivational systems organizing child’s development, specifically provides fear regulation mechanisms and thus shapes the child’s ability to regulate her/his emotions. The purpose of the attachment system is indeed promoting and maintaining the secure-base (care giving adult) proximity in order to provide secure intrapsychic processes (normal emotional regulation).

Another key feature of the attachment theory is its inherent relational disposition (bonding). An “attachment bond” (Bowlby and Ainsworth 1992) is a peculiar kind of *relational stance*, one where the infant looks for a protective relationship with the “older and wiser” adult, i.e. a caregiver. The individual differences in organisation of attachment behaviour (called attachment organisation or attachment style) amongst infants are most often classified as Secure, Insecure (Anxious-Avoidant or Anxious-Resistant) and Disorganised (Ainsworth 1979, 1978; Bowlby 1988). Attachment is “secure” if the child receives protection and security through early developmental stages, and the relationship between the child and his/her caregiver(s) becomes successful throughout life. This implies that the caregiver must provide some basic conditions that are essential for a responsive relationship in order to regulate fear (child’s fear) when attachment system is activated. These basic requirements include emotional availability, sensitivity and empathic attunement; all of which must be maintained through various life phases; or as Bowlby and Ainsworth (1992) puts it, from “the cradle to the grave”.

In other words, attachment organisations and styles are found in adults where early childhood experiences set the precedent or draw a road map for later adult attachment behaviours (see Noftle and Shaver 2007; Bartley 2007; Mikulincer and Shaver 2007). Although factor structures arising from different adult attachment studies differ slightly, most adult attachment measures do show a factor analysis that is linked to the theoretical model of Secure, Insecure and Disorganised styles (Mikulincer and Shaver 2007). The main implication of this finding is that people organize their emotion regulation and primary relational modalities in a fairly constant way over the life span due to the development of “internal working models” of relationships (Bowlby 1988). In short, the seminal construct of I.W.Ms. (Internal Working Models) refer to the building up of progressively more mature and sophisticated attachment interactions, mental representations and correlated expectations that allow the child to predict and reflect upon supportive relationships later in life. For example, the more the child experiences secure attachment; the expectations will be more articulated, open, benevolent and positive relational experiences in adulthood. In addition, while the I.W.Ms. affect romantic and other types of relationships, these mental representations also play a major role in parenthood. In the light of the inter transmission constructs, there tends to be a positive correlation between attachment status in the caregiver and the child, especially where the caregiver utilises secure attachment styles.

Moreover, current evidence supports a cross-generational transmission risk of developing depression from mother to child, particularly during the offspring’s childhood and adolescence periods (Bureau et al. 2009); with female trans-generational transmission of PND posing significant challenges to healthcare providers (Sejourne et al. 2011). The links between PND and many different adverse health outcomes for children are strong when depression is chronic and the family has low socioeconomic status (Murray and Cooper 1997; Kurstjens and Wolke 2001; Parsons et al. 2012). Children of depressed mothers have been found to attend fewer health clinics, receive fewer vaccinations and receive more emergency health care interventions than those of mothers with no mental health problems (Minkovitz et al. 2005; Alhusen et al. 2013; Kohlhoff and Barnet 2013). The emotional development of the child is impacted by the mother’s depressive status, which in turn can lead the child to develop cognitive deficits (Beck 1998) and behavioural problems (Bagner et al. 2010), which also affects the child’s academic achievements (Hay et al. 2001). In some extreme cases, PND outcomes can include suicide and infanticide, though infanticide is less common than suicide among mothers with PND conditions (Spinelli 2004).

This systematic review aims to gain more in-depth understanding of the link between PND and adult attachment styles. Particularly, we aim to systematically analyse and discuss the extent to which the adult attachment style of a new mother can be conceptualised as a risk factor for the development of postnatal depression.

## Method

We carried out a systematic search into the current literature using the following databases: PsycInfo, PsycExtra Web of Science, The Cochrane Library and Pubmed. We focused on research papers that examined adult attachment styles and PND, and published between 1991 and 2013. Search terms used were as follows: Attachment, Adult, Maternal, Postnatal, Postpartum, Puerperium and Depression. The final searches in each database took the following form: *(Adult OR Maternal] AND Attachment) AND ([Postnatal OR Postpartum OR Puerperium] AND Depress). We included any papers showing relationship between maternal adult attachment and PND.*

See Table 1 for more information on inclusion criteria for selecting relevant research papers for the final review. Papers were excluded if they were studying maternal-infant attachment and PND (not maternal adult attachment). We also excluded papers if they did not include both a depression measure and an adult attachment measure or because measures were taken either prenatal or after 12 months postpartum. We assessed the quality of the studies examining the associations between attachment style and PND, adapting the Effective Public Health Practice Project (EPHPP) Quality Assessment Tool, together with the quality assessment Tables we previously compiled, making the revised manuscript stronger (see Table 2). No papers received a strong rating for sampling as all samples were conveniently obtained; geographically limited and/or were clinical samples (rather than sampled from general populations).

**Table 1:** **Inclusion and exclusion criteria**

**Limits: Human studies, English language, 1991-2013**	
**Inclusion criteria**	**Exclusion criteria**
*Studies concerning the mother’s own adult attachment style*.	*Studies specifically involving premature infants.*
*Papers published from 1991–2013.*	*Media outside of academic journals.*
*Journal articles only.*	*Studies investigating male PND or couple’s PND where data is not grouped according to Gender.*
*Studies using a depression scale and an adult attachment scale*	*Studies investigating depression linked to still births.*
S*tudies concerning assisted vs. natural conception and typical vs. atypical pregnancies and births.*	*Investigations of the attachment between mother and infant. *This study concerns the mothers own adult attachment style only, not the bidirectional attachment of between infant and mother.
*Studies concerning adult and adolescent mothers.*	*Studies assessing participants with known pre-existing psychiatriccomorbidities.*
*Quantitative data*	*Maternal depression occurring after the first year of motherhood.*

**Table 2:** **Quality assessment table**

**Quality assessment**	**Hypothesis**	**Outcome measures**	**Sampling**	**Confounders**	**Cause-effect associations**	**Other associations**
Akman et al. 2006	Y	Y	M	M	W	S
Akman et al. 2008	Y	Y	M	M	W	S
Besser et al. 2002	Y	Y	M	M	W	S
Bifulco et al. 2004	Y	Y	M	M	W	S
Conde et al. 2011	Y	Y	M	M	W	S
Flykt et al. 2010	Y	Y	M	M	W	W
Kuscu et al. 2008	Y	Y	M	M	W	S
McMahon et al. 2005	Y	Y	M	M	W	S
Meredith and Noller 2003	Y	Y	M	M	W	S
Monk et al. 2008	Y	Y	M	M	W	S
Pesonen et al. 2004	Y	Y	M	M	W	S
Sabuncuoglu and Burkem 2006	Y	Y	M	M	W	S
Scharfe 2007	Y	Y	M	M	W	S
Simpson et al. 2003	Y	Y	M	M	W	S
Koholhoff and Barnet 2013	Y	Y	M	M	W	S
Alhusen et al. 2013	Y	Y	M	M	W	S
Wilkinson and Mulcahy 2010	Y	Y	M	M	W	S
Wilkinson and Scherl 2006						

Y = yes; M = moderate’ W = weak; S = strong.

A strong or moderate rating was given when there was a control group, or the design was longitudinal. A study given strong or moderate rating would have given good validity and reliability values for all measures. A study was assigned weak or strong scores for reporting (or not) direct cause-effect correlations, or moderate rating if an association was made with no specified directions (e.g., cross-sectional). After titles and abstracts were screened from databases such as Pubmed (112), Web of Science (242) and Psycinfo (115), whilst removing non-relevant articles from other sources, 28 articles we carried forward for further examination. 20 Articles met inclusion and criteria, and were selected for the final review. See Additional file 1 for adapted PRISMA flowchart.

### Data analysis

The design of this review is a systematic review with narrative synthesis, although reporting on statistical data when it is appropriate (meta-analysis). This method was selected having carried due to the heterogeneity of measurement tools used in the resulting studies and that the research question does not concern efficacy of any treatments for PND. Out of 353 papers, 20 met the study inclusion criteria, representing a total of 2306 participants. Data from these 20 studies was extracted by means of a data extraction table. Tables 2 and 3 summarises the setting of each study and its participants, study design, measures used, aim or hypothesis, quality rating and whether or not a link was found between adult attachment style and PND. The sample represents parents from a range of locations, the majority of these being western countries, but cohorts from Israel (Besser et al. 2002) and Turkey (Akman et al. 2006, 2008; Kuscu et al. 2008; Sabuncuoglu and Berkem 2006) were also found. Six studies carried out hypothesis testing (Besser et al. 2002; Feeney et al. 2003; Kuscu et al. 2008; Meredith and Noller 2003; Pesonen et al. 2004; Simpson et al. 2003), the rest of the studies had no direct hypotheses, only examining associations between PND and adult attachment in the same study or as party of a larger study. Six were cross-sectional (Kuscu et al. 2008; Meredith and Noller 2003; Pesonen et al. 2004; Sabuncuoglu and Berkem 2006; Wilkinson and Mulcahy 2010; Wilkinson and Scherl 2006) and the other 14 were longitudinal with varied follow-up lengths of between 8 weeks (Besser at al 2002) and 12 months postpartum (McMahon et al. 2005).

**Table 3:** **Data extraction table**

**Author & year**	**Location**	**Participants**	**Design & length of postnatal follow up**	**Measures used for depression and attachment**	**Quality rating**	**Hypothesis (H) or aim of study**	**Was attachment style found to be significantly associated with PND?**
Akman et al. 2006	Turkey	78 women and infants from a hospital maternity department.	Q-e.	EPDS (self-report) AAS (self-report)	50%	Aim: investigate the frequency of and relationship between infant colic, and maternal attachment styles, depression and anxiety.	Yes
			L. 4–6 months				
Akman et al. 2008	Turkey	60 women from a hospital maternity department.	Q-e.	EPDS (self-report) AAS (self-report)	61%	Aim: examine the adjustment of mothers (depression, anxiety, support and maternal attachment styles) in the context of breastfeeding.	Yes
			L. 4 months				
Besser et al. 2002	Israel	200 mothers from 10 well-baby clinics.	Q-e.	CES-D (self-report) RQ (self-report)	89%	H1: Positive other models and support will predict lower depression.	Yes
			L. 8 weeks			H2: Secure maternal attachment will correlate with low levels of depression.	
						H3: Social support and maternal attachment will moderate depression.	
Bifulco et al. 2004	Europe* and USA	204 women from antenatal clinics or classes (and a comparison group of 80 women from GP practices in London in the 1990s)	Q-e.	SCID-PND (Interview) ASI (Interview)	79%	Aim: Develop the SCID-PND and its associations with social contexts and depression.	Yes
			L. 6 months				
Conde et al. 2011	Portugal	63 couples from an antenatal obstetric unit in a maternity hospital.	Q-e.	EPDS (self-report) ASI (Interview)	80%	Aims: Examine the effects of attachment style and partner support in men and women on depression and anxiety symptoms pre and postpartum.	Yes
			L. 3 months				
Feeney et al. 2003	Australia	150. 76 “transition” and 74 “comparison” couples from the university psychology participant pool, media releases and relevant health care settings e.g., antenatal clinics.	Q-e.	Short-Form Depression Anxiety Stress scales (self-report) ASQ (self-report)	88%	H1: Attachment will be less stable for transition group wives.	Yes
			L. 6 months				
						H2: Relationship anxiety will predict increased depression, more so for those reporting husbands as less supportive.	
						H3: Maternal depression will be associated with higher attachment insecurity and relationship dissatisfaction for both husbands and wives.	
Flykt et al. 2010	Finland	49 mothers and their children from maternity health-care centres.	Q-e.	EPDS (self-report) AAI Questionnaire (self-report)	69%	H1: Pre and postnatal depression effect mother-child relationship dyads.	No
			L. 4–5 months				
						H2: Secure maternal attachment protects the dyadic interaction from harmful effects of depression.	
Kuscu et al. 2008	Turkey	100 mothers from a hospital maternity department.	Q-e.	EPDS (self-report) AAS (self-report)	61%	Aim: to evaluate the predictors of depressive symptoms associated with childbirth emphasising maternal attachment and family support.	Yes
			C-s.				
McMahon et al. 2005	Australia	100 women from a parent-craft centre (for support with infant difficulties e.g. feeding, sleeping and settling).	Q-e.	CIDI-D (Interview), CES-D (self-report)	56%	Aim: to explore predictors of persistence of PND at 12 months, particularly adverse childhood experience, and the mediating effects of current interpersonal difficulties.	Yes
			L. 12 months	ASQ (self-report)			
Meredith and Noller 2003	Australia	72 mothers from media releases or a maternity hospital (n = 38) or from a residential facility for mothers with child-related concerns e.g., feeding, sleeping and behavioural difficulties (n = 36).	Q-e.	EPDS (self-report)	66%	H1: Women with PND more likely to have insecure attachment styles.	Yes
			C-s.	RQ (self-report)		H2: Mothers reporting PND will perceive infants as more difficult and have less positive relationships with their partners.	
						H3: Mothers reporting insecure attachment will report less positive relationships with both her child and her partner.	
Monk et al. 2008	North East U.S	56 mothers from posted announcements and signs in obstetricians’ offices.	Q-e.	CES-D (self-report)	86%	Aim: Investigate the link between attachment style and pregnancy experience and perinatal and postnatal depression.	Yes
			L. 4 months	RSQ (self-report)			
Pesonen et al. 2004	Finland	319 mothers, 319 infants and 173 fathers from a large maternity hospital.	Q-e.	CESD- 10 (self-report)	70%	H1: Secure adult attachment operates as a buffer between depressive symptoms and negative perception of infant temperament.	Yes
			C-s.	AAS and RQ (both self-report)			
Sabuncuglu and Berkem 2006	Turkey	80 women from a mother-infant health care centres providing services for low-moderate income families.	Q-e.	EPDS (self-report)	65%	Aim: Explore the relationship between PND and insecure attachment style in Turkish mothers.	Yes
			C-s.	AAQ (self-report)			
Scharfe 2007	Canada	235 women from a hospital prenatal clinic.	Q-e.	EPDS (self-report)	56%	Aim: Investigate the causal relationships between attachment models and depression.	Yes
			L. 6 months	RSQ (self-report)			
Simpson et al. 2003	South-west U.S	99 married couples from a childbirth course.	Q-e.	CES-D (self-report)	61%	Ambivalent women perceiving their husbands as angry (H1) or less supportive (H2) will have increases in postnatal depressive symptoms. These perceptions will not affect avoidant women (H3). Changes in these perceptions will mediate the above interaction terms (H4 and H5).	Yes
			L. 6 months	AAQ (self-report)			
Wilkinson and Mulcahy 2010	Australia	115 (Likely to be depressed n-47 and comparison group n = 68) women from health-care professional referrals.	Q-e.	EPDS (self-report)	74%	Aim: Clearly establish links between attachment models, PND and other social adjustment indicators.	Yes
			C-s.	RQ (self-report)			
Wilkinson and Scherl 2006	Australia	60 mothers from baby health and immunisation clinics and snowball sampling.	Q-e.	EPDS (self-report)	40%	Aim: to explore the psychological health and attachment styles of breast and formula feeding mothers.	Yes
			C-s.	RQ (self-report)			
Alhusen et al. 2013	East Coast	81 follow up mothers from a previous cross-sectional study	L. 9 months	EPDS (Interview)	85%	Women with an insecure attachment style would (a) have had lower MFA during pregnancy and (b) higher depressive symptomatology in the post-partum period.	Yes
	US			ASQ			
Kohlhoff and Barnet 2013	Australia	83 primiparous women	C-s	EPDS (Interview)	85%	Maternal depression would mediate the relations between adult attachment insecurity and parenting self-efficacy	Yes
				ASQ			

## Results

Every paper in this review found adult attachment style to be significantly linked to PND symptoms, with the exception to Flykt et al. 2010. Seven studies reported an anxious style being significantly linked to PNDS (Bifulco et al. 2004; Feeney et al. 2003; Kuscu et al. 2008; McMahon et al. 2005; Meredith and Noller 2003; Scharfe 2007; Simpson et al. 2003) whereas only three (Monk et al. 2008; Wilkinson and Mulachy 2010) found avoidant styles were more predictive of PNDS. Eight studies found both anxious and avoidant or simply ‘insecure’ styles to be related to maternal PND (Akman 2006; Besser 2002; Conde et al. 2011; Kohlhoff et al. 2013; Pesonen et al. 2004; Sabuncuoglu and Berkem 2006; Alhusen et al. 2013; van Bussel et al. 2005. When comparing studies with regards to the self-report vs. interview conceptualisations of adult attachment, it is difficult to place the Flykt et al. (2010) study into either conceptual school of thought regarding adult attachment because it used the AAI (an interview measure) which had been converted into a questionnaire (self-report). The validity of this measure is not clear and it presents a complication when trying to explain the reason behind the lack of association between the two variables in question. The only other interview measure used across the studies, the Attachment Style Interview, was used by Bifulco et al. (2004) and Conde et al. (2011) and did give rise to a significant association between mother’s insecure attachment and PND. Self-report depression measures were also more frequently used, with only two studies using diagnostic interviewing (Bifulco et al. 2004; McMahon et al. 2005). Table 4 show statistically significant variables that are linked to PND (**) or Attachment style (*).

**Table 4:** Variables/demographics analysed in studies and whether they were recorded to be significantly linked to PND (**) or attachment style (*)

**Variables noted in study**	**Study (numbered according to referencing)**																			
	**1**	**2**	**3**	**4**	**5**	**6**	**7**	**8**	**9**	**10**	**11**	**12**	**13**	**14**	**15**	**16**	**17**	**18**	**19**	**20**
**Maternal age**	X	X	X*	X	X	X	X	X	X	X	**X		X	X	X	**X	X	X	X	X
**Employment status**	X	X		X*	X	X		X					X			X	**X		X	X
**Housewife or primary carer**	X	X				X		X			X		**X	X			X			
**Income**					X		X			**X	**X								X	
**Social class**				X*	X		X											X		
**Education level**	X	X	X	X	X	X	X	X	**X		**X	X		X		X		X	X	
**National language proficiency**									**X											
**Marital status**			X	X*	X	X	X			X	X			X	X	X	X		X	
Relationship quality or satisfaction						X			**X	X*			X		X*		**X*			
Spousal behaviours or support			**X*		X	X				**X					**X*					X
Spouse attachment style					X*	X*									X*					
Spouse depression						**X														
Husband happiness re: pregnancy										**X										
Extended family				X*	X			**X												X
Social/family support		X*		X*		X		**X									**X*			
Historic parental separation				**X*				X												
Early childhood experiences of parents								X	**X*	**X						**X			**X*	X
Planned pregnancy						X		X		**X			X			X		X		
Pregnancy experience						X					**X*					**X		X		
Parity		X		X	X		X			**X	X		X	X	X	X	X	X		
Gestational Diabetes			**X																	
**Age of infant**							**X			X			X				**X	X		
Breastfeeding duration		**X											X					X*		
Infant health problems										**X										X
Infant colic	**X*																			
Perceived infant temperament or difficultness						**X				**X		**X*								
Mother-infant interaction							**X*													
Infant-mother attachment																	**X*	X		
Prenatal depression			**X	X*	X	X	X				**X				X	**X			X*	
History of depression										**X	X						**X			X
Life adversity/stress								X			**X*									X
Psychological wellbeing								X										**X*		
Maternal Anxiety	X*	X			X*	X		**X			**X							**X*	**X*	X
Maternal Neuroticism															X*	**X				
Cognitive defence/coping style									**X							**X				

Variables in bold type are considered to be demographics.

Study numbers in bold type are those that included demographics in the inferential statistical analysis.

**Significant link to depression findings.

*Significant link to attachment findings.

No asterisk indicates findings linking to attachment or depression, or that it was not analysed statistically.

Note that links may be from correlation analysis, tests of group differences or modelling analyses.

This Table also shows demographic and other relevant data included in the inferential statistical analyses of the 20 studies. Three variables were found to be linked only to attachment style in three separate studies; social class (Bifulco et al. 2004), marital status (Bifulco et al. 2004), and spouse attachment style (Conde et al. 2011; Feeney et al. 2003; Simpson et al. 2003). Ten variables were associated with PND score alone and each in one study only; infant health problems (Meredith and Noller 2003), gestational diabetes (Besser et al. 2002), parity, planned pregnancy, partner’s happiness regarding the pregnancy (Meredith and Noller 2003), national language proficiency (McMahon et al. 2005) and being the primary caregiver/a housewife (Sabuncuoglu and Berkem 2006). Age of infant, (Flykt et al. 2010; Wilkinson and Mulachy 2010) education level (McMahon et al. 2005; Monk et al. 2008), perceived infant temperament (Feeney et al. 2003; Meredith and Noller 2003), income (Meredith and Noller 2003; Monk et al. 2008), early childhood experience of parents (Meredith and Noller 2003; Alhusen 2013; Van Bussel et al. 2005), cognitive defence/coping style (McMahon et al. 2005; Van Bussel et al. 2005) and history of depression (Meredith and Noller 2003; Wilkinson and Mulachy 2010) were each found to be linked to PND symptoms in two separate studies, increasing the validity of these findings.

Variables found to be correlated to both PND symptoms and attachment style but over separate studies were maternal age (Besser et al. 2002; Monk et al. 2008; and Van Bussel et al. 2005), employment status (Bifulco et al. 2004; Wilkinson and Mulachy 2010), relationship quality/satisfaction (McMahion et al. 2005, Meredith and Noller 2003; Scharfe 2007 and Wilkinson and Mulachy 2010), living with extended family (Bifulco et al. 2004; Kuscu et al. 2008), social support (Akman et al. 2008; Bifulco et al. 2004; Kuscu et al. 2008; Wilkinson and Mulachy 2010), breastfeeding duration (Akman et al. 2008; Wilkinson and Scherl 2006), prenatal depression (Besser et al. 2002; Bifulco et al. 2004; Monk et al. 2008; Van Bussel 2005), maternal anxiety (Akman et al. 2006; Conde et al. 2011; Kuscu et al. 2008; Monk et al. 2008) and maternal neuroticism (Simpson et al. 2003 and Van Bussel et al. 2005).

Risk factors associated with both PND symptoms and attachment style in the same study analysis were maternal anxiety and psychological wellbeing (Wilkinson and Mulachy 2010; Kohlhoff et al. 2013), life adversity/life stress (Monk 2008), infant to mother attachment (Alhusen et al. 2013; Flykt et al. 2010), perceived infant temperament (Pesonen et al. 2004), infantile colic (Akman et al. 2006), experience of pregnancy (Alhusen et al. 2013; Monk et al. 2008), early childhood experience of parents (Alhusen et al. 2013; McMahon et al. 2005), historic parental separation during childhood (Bifulco et al. 2004), social support and relationship quality/satisfaction (Wilkinson and Mulachy 2010) and spousal behaviour/support (Besser et al. 2002 and Simpson et al. 2003).

## Discussion

Depression is one of the most widely studied clinical conditions, particularly when it comes to maternal depression and child attachment security. Maternal PND is a clinical condition that not only affects the mother but also the psychological health of the whole family (NICE, 2007). Children of depressed mothers are at higher risk to develop insecure attachments as maternal depression tends to affect the quality and sensitivity of parenting styles. This process has been demonstrated in relation to the more transient form of maternal depression, i.e. PND. Several authors illustrate how the infant bonding process can be threatened by maternal PND, which in turn leads to less secure infant attachment experience and more disorganised (van IJzendoorn 1999; Martins and Gaffan 2000), as well as avoidant infant attachment styles (Alhusen 2013; Kohlhoff 2013). 19 out of 20 studies included in this review found PND to be statistically associated with insecure adult attachment style. Compared with avoidant or dismissing styles, anxious attachment styles were more frequently associated with PND symptoms, although this may be merely due to the defensive characteristic that avoidant adults tend to exhibit.

The findings of this systematic review show that other risk factors were related to both PND and insecure adult attachment style (see Table 4); hinting at a covariance between these two constructs (See for example, Kohlhoff et al. 2013). In the case of early loss of attachment relationship and/or in consistent secure attachment provision from a primary caregiver, the child is at a higher risk for developing an insecure attachment style later in life, with an associated risk to the onset of a PND condition that predicts depression during childhood and adolescence stages (Duggal et al. 2001). Referring back to the roles played by the Internal Working Models in parenthood, mother’s PND will affect her child’s attachment style through concomitant impairment of sensitive and inadequately attuned care giving practices. In other words, the adverse life events experienced by a mother with PND will have a knock on effect on her child, both through the mother’s clinical conditions and because of not being able to promote and maintain a stable and secure attachment relationship. Recently, the UK Patients’ Association used the Freedom Information Act to obtain information from 150 Primary Care Trusts (PCTs) on NHS service provision for mothers with PND conditions. Their report found that *78% of the PCTs have no information of the incidence of PND in their region; 45% failed to provide information on the number of “Serious Untoward Incidents” related to PND in the services they commission, and 55% of PCTs do not provide information to mothers with PND* (The Patients Association, 2011; Pg1). The evidence-based information pulled together in this systematic review will enable practitioners and researchers to have a better understanding of the epidemiology and predictors of postnatal depression, with insecure attachment style and a range of other adverse factors complicating the specific needs of this patient group. Comparatively, a few studies have investigated direct cause-effect links between adult attachment style and PND, again highlighting the importance of acknowledging and addressing PND and its relation to attachment style as an emerging public health priority for the general population (including adult groups); both from research and clinical perspectives.

### Methodological considerations

A review of research is only as great as the sum of its parts, even if the outcomes of the studies are pointing towards the same conclusion. One is not able to ascertain whether this data is generalizable as the reliability of the samples implying population level results is restricted. Measurement of depression varied across the studies in different ways. Measurement tools used were not uniform (see data extraction Table) and the type of data used in analysis varied. Only one study analysed depression data as categorical data, depressed or not depressed; the rest of studies featured analysis of depression as continuous or both and categorical, meaning the depression was understood as a common sub-threshold symptom that was relevant even if clinical diagnoses were not made. Cut off scores for depression varied across studies, for example, of the 13 studies using the EPDS (see Table 3), two used continuous data only and four studies used a cut off score of ≥13, three used a cut off score of ≥12, one used a cut off score of ≥11 and one used a cut off score of ≥10. For these reasons, it is important to note that this review uses the term PND (post natal depression symptoms) when making conclusions informed by the data from these 20 studies.

Moreover, there was no data on adult attachment styles and PND in low income countries, and therefore difficult to know if mothers living in low income countries experience PND conditions, induced by insecure adult attachment styles. A key recommendation for future research would be to complete large international longitudinal studies from multiple selection sites, using a mixture of interview and self-report attachment and PND measures in order to redress the imbalance of white, middle class, western, married participants within this research pool. It would be important to measure and model those variables such as self-esteem and family history of mental illness which may also explain some of the shared variance in PND and attachment scores, which is missed by the current literature. The use of tools as screening devices for empirically established risk factors for postnatal depression is helpful in antenatal and mental health care systems. This will allow for tailoring care to the individual formulation of risk and could provide healthcare workers and prospective mothers with knowledge, psycho-education and more resources to intervene if PND should develop.

## Conclusions

Although no cause-effect direction has been established yet, one can theoretically deduce the direction of the relationship. Because attachment is a trait which develops in infancy and is fairly stable across the lifespan, insecure adult attachment should be highlighted as a highly important risk factor for PND. Factors linked to adult attachment style include low economic status, being female, experiencing childhood adversity (trust betrayal, abuse and/or loss), parental psychopathology and neuroticism. These variables are also implicated as causing PND. Other risk factors of PND linked to adult attachment style are: lower opinions of partner support (Rholes et al. 2001), lower levels of marital relationship functioning (Rholes et al. 2001), lower levels of relationship satisfaction (Brennan and Shaver 1995; Shi 2003), and social support (Anders and Tucker 2000). Finally, the development and use of a specific measure that taps on adult attachment and PND measure could prove an effective way to formulate ones’ risk, to help prepare the family for the arrival of the child and for services to funnel resources and time into those families who are more at risk of insecure adult attachment style and PND conditions.

## Additional file

Additional file 1:
**Flow chart (Adapted from PRISMA 2009 Flow Diagram).**
