# Supplementary material for: Adult attachment style as a risk factor for maternal postnatal depression: a systematic review
Source: BMC Psychol. 2014 Dec 18;2(1):56. doi: 10.1186/s40359-014-0056-x (PMC4407393; doi:10.1186/s40359-014-0056-x)
Supplement: Additional file 1: — Flow chart (Adapted from PRISMA 2009 Flow Diagram). [file 40359_2014_56_MOESM1_ESM.doc]

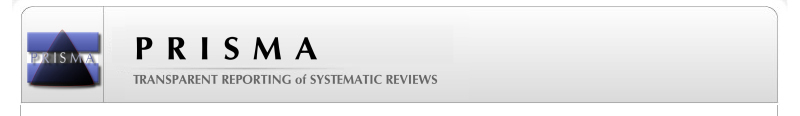
 **Flow Chart (Adapted from PRISMA 2009 Flow Diagram)**

**Screening**

**Included**

**Eligibility**

**Identification**

Records identified through database searching
(n = 486)

Records screened after duplicates
(n = 353)

Full-text articles assessed for eligibility
(n = 28 )

Full-text articles excluded, (n = 8)

Studies included in qualitative synthesis
(n = 20)

Studies included in quantitative synthesis (meta-analysis)
(n = 20)
